# Supplementary material for: Acute and Sub-Chronic Effects of Microplastics (3 and 10 µm) on the Human Intestinal Cells HT-29
Source: Int J Environ Res Public Health. 2021 May 28;18(11):5833. doi: 10.3390/ijerph18115833 (PMC8198674; doi:10.3390/ijerph18115833)
Supplement: Supplementary file 1 [file ijerph-18-05833-s001.zip › ijerph-1179470-supplementary.pdf]

Supplementary material

Figure S1: PS-MPs 3 and 10 μm suspensions in cell medium after 24h and 48h.

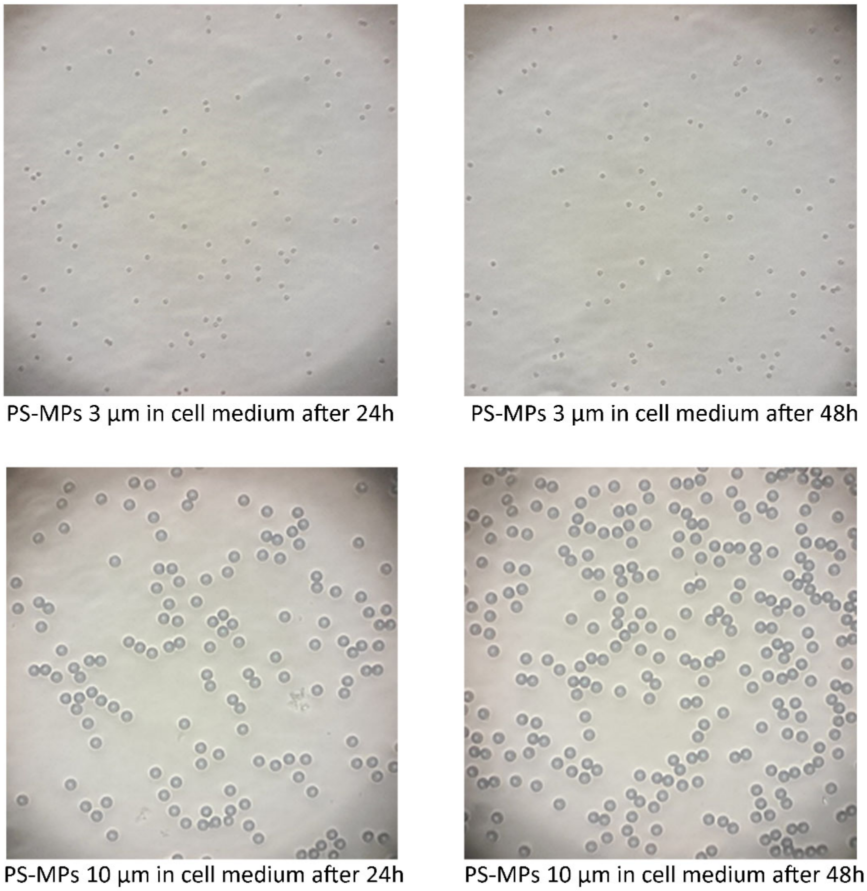

To minimize PS-MPs agglomerate in liquid, due to their hydrophobicity and large surface area, the stock microplastic suspensions were sonicated for 20 min (frequency 40 kHz) prior to be added to the medium. Preliminarily microscopic observations were performed to assess if the PS-MPs were uniformly dispersed in cell medium or if, on the contrary, they formed aggregates.

The figure S1 clearly shows that in cell medium neither of the used PS-MPs formed aggregates after 24h and 48h of incubation, remaining well separated up to the concentration of 160,000 p mL^{-1} for both particles.
